# Supplementary material for: Maternal Blood Levels of Toxic and Essential Elements and Birth Outcomes in Argentina: The EMASAR Study
Source: Int J Environ Res Public Health. 2022 Mar 18;19(6):3643. doi: 10.3390/ijerph19063643 (PMC8954125; doi:10.3390/ijerph19063643)
Supplement: Supplementary file 1 [file ijerph-19-03643-s001.zip › ijerph-1630999-supplementary.pdf]

Supplementary materials

for

Maternal Blood Levels of Toxic and Essential Elements and Birth Outcomes in Argentina. The EMASAR study

Shanshan Xu [1,2], Solrunn Hansen [3], Kam Sripada [4,5], Torbjørn Aarsland [6], Milena Horvat [7], Darja Mazej [7], Marisa Viviana Alvarez [8] and Jon Øyvind Odland [1,9,*]

[1] Department of Public Health and Nursing, Norwegian University of Science and Technology (NTNU), 7491 Trondheim, Norway

[2] Center for International Health, Department of Global Public Health and Primary Care, University of Bergen, 5009 Bergen, Norway; shanshan.xu@uib.no

[3] Department of Health and Care Sciences, UiT The Arctic University of Norway, 9037 Tromsø, Norway; solrunn.hansen@uit.no

[4] Centre for Global Health Inequalities Research (CHAIN), Norwegian University of Science and Technology (NTNU), 7491 Trondheim, Norway

[5] Centre for Digital Life Norway, Norwegian University of Science and Technology (NTNU), 7491 Trondheim, Norway; kam.sripada@ntnu.no

[6] Research Department, Stavanger University Hospital, 4068 Stavanger, Norway; toaa@lyse.net

[7] Department of Environmental Sciences, Jožef Stefan Institute, 1000 Ljubljana, Slovenia; milena.horvat@ijs.si; darja.mazej@ijs.si

[8] Hospital Público Materno Infantil de Salta, Sarmiento 1301, 4400 Salta, Argentina; marisavivianaalvarez@gmail.com

[9] School of Health Systems and Public Health, Faculty of Health Sciences, University of Pretoria, Pretoria 0002, South Africa; jon.o.odland@ntnu.no

Corresponding author:

Jon Øyvind Odland

Department of Public Health and Nursing,

Norwegian University of Science and Technology, 7491 Trondheim, Norway

E-mail address: jon.o.odland@ntnu.no

Table of contents

**Table S1.** Descriptive statistics of whole blood elements levels (μg/L) among delivering women in the overall studied population	Page 3
**Table S2.** Results of multiple linear regression models showing effects of socio-economic determinants in the blood levels of toxic and essential elements	Page 4
**Table S3.** Multiple linear regression of maternal elements levels and birth outcomes. Changes in birth outcome per unit increase in ln-transformed elements concentrations (μg/L) in the overall samples are presented	Page 5
**Table S4.** Regional-specific multiple linear regression of maternal elements levels and birth outcomes	Page 6
**Table S5.** Results of logistic regression analyses showing the relationships between tertile of blood elements levels and preterm birth and low birth weight	Page 7
**Figure S1.** Directed acyclic graph for gestational age model	Page 8
**Figure S2.** Directed acyclic graph for birth weight, birth length and head circumference models	Page 8
**Figure S3.** Directed acyclic graph for preterm birth model	Page 9
**Figure S4.** Directed acyclic graph for low birth weight model	Page 9

**Figure S5.** Spearman's correlation coefficients (ϱ) among the whole blood toxic and essential elements stratified by study sites	Page 10
**Figure S6.** Spearman's correlation coefficients (ϱ) between maternal elements concentrations among the overall postpartum women in the EMASAR study	Page 11

Table S1. Descriptive statistics of whole blood elements levels (μg/L) among delivering women in the overall studied population (n = 696).

Essential and toxic elements	Limit of detection	GM (95%CI)	Min-Max	Selected percentiles		
				25th	50th	75th
Cu	5	1754 (1734-1775)	961-3559	1598	1748	1943
Mn	0.5	21.38 (20.88-21.89)	4.75-52.96	17.78	21.70	26.73
Se	5	112.79 (110.48-115.14)	51.28-240.57	92.04	118.68	137.95
Zn	30	6940 (6857-7023)	4171-10199	6192	6920	7828
As	0.1	0.57 (0.56-0.59)	0.22-3.78	0.45	0.54	0.67
Cd	0.05	0.19 (0.18-0.20)	0.04-1.05	0.14	0.19	0.26
Hg	0.2	0.52 (0.50-0.56)	0.15-19.58	0.32	0.51	0.85
Pb	1	13.93 (13.41-14.46)	2.94-152.31	10.03	13.40	18.51

Abbreviations: As, arsenic; Cd, cadmium; Cu, copper; Hg, mercury; Mn, manganese; Pb, lead; Se, selenium; Zn, zinc; GM, geometric mean; CI, confidence interval.

Table S2. Results of multiple linear regression models showing effects of socio-economic determinants in the blood levels of toxic and essential elements (n = 636).

Essential elements	Variable	Std. β[a]	*P* value	Toxic elements	Variable	Std. β[a]	*P* value
**Cu**	Age, years	0.11	**0.025**	**As**	Age, years	0.02	0.733
	Pre-pregnancy BMI, kg/m^2	0.08	0.058		Pre-pregnancy BMI, kg/m^2	-0.09	**0.032**
	Parity, P1-MP[b]	-0.13	**0.006**		Parity, P1-MP[b]	0.06	0.208
	Smoking, no-yes[c]	-0.02	0.570		Smoking, no-yes[c]	0.03	0.497
	Education, low-high[d]	-0.10	**0.027**		Education, low-high[d]	0.04	0.405
	Study site, Salta-Ushuaia[e]	-0.12	**0.006**		Study site, Salta-Ushuaia[e]	0.14	**0.001**
	Residence, urban-rural[f]	0.04	0.264		Residence, urban-rural[f]	0.02	0.701
**Mn**	Age, years	-0.12	**0.010**	**Cd**	Age, years	0.30	**0.001**
	Pre-pregnancy BMI, kg/m^2	0.05	0.188		Pre-pregnancy BMI, kg/m^2	-0.15	**< 0.001**
	Parity, P1-MP[b]	0.14	**0.001**		Parity, P1-MP[b]	0.05	0.308
	Smoking, no-yes[c]	-0.04	0.250		Smoking, no-yes[c]	0.16	**< 0.001**
	Education, low-high[d]	-0.13	**0.002**		Education, low-high[d]	-0.06	0.147
	Study site, Salta-Ushuaia[e]	-0.28	**< 0.001**		Study site, Salta-Ushuaia[e]	-0.1	**0.035**
	Residence, urban-rural[f]	-0.02	0.584		Residence, urban-rural[f]	0.03	0.402
**Se**	Age, years	0.003	0.923	**Hg**	Age, years	0.03	0.565
	Pre-pregnancy BMI, kg/m^2	0.01	0.795		Pre-pregnancy BMI, kg/m^2	0.004	0.916
	Parity, P1-MP[b]	0.11	**< 0.001**		Parity, P1-MP[b]	-0.06	0.172
	Smoking, no-yes[c]	-0.002	0.931		Smoking, no-yes[c]	-0.06	0.158
	Education, low-high[d]	-0.04	0.171		Education, low-high[d]	0.07	0.104
	Study site, Salta-Ushuaia[e]	-0.78	**< 0.001**		Study site, Salta-Ushuaia[e]	-0.39	**< 0.001**
	Residence, urban-rural[f]	-0.04	0.110		Residence, urban-rural[f]	0.04	0.300
**Zn**	Age, years	0.02	0.600	**Pb**	Age, years	0.1	**0.029**
	Pre-pregnancy BMI, kg/m^2	-0.03	0.491		Pre-pregnancy BMI, kg/m^2	0.03	0.438
	Parity, P1-MP[b]	0.15	**0.001**		Parity, P1-MP[b]	0.03	0.489
	Smoking, no-yes[c]	-0.03	0.434		Smoking, no-yes[c]	0.09	**0.016**
	Education, low-high[d]	-0.03	0.493		Education, low-high[d]	-0.06	0.139
	Study site, Salta-Ushuaia[e]	0.37	**< 0.001**		Study site, Salta-Ushuaia[e]	-0.41	**< 0.001**
	Residence, urban-rural[f]	-0.07	0.060		Residence, urban-rural[f]	-0.05	0.183

Bold *p* values are statistically significant ($p < 0.05$).
[a] Standardized regression coefficients β.
[b] Para 1 *vs.* multiparous. Para 1 as reference category.
[c] Non-smoker *vs.* smoker. Non-smoking women as reference.
[d] Primary and secondary education (low education) *vs.* Tertiary and university education (high education). Women with primary and secondary education as reference.
[e] Salta *vs.* Ushuaia. Salta as the reference site.
[f] Urban *vs.* rural. Urban dweller as reference category.
Abbreviations: As, arsenic; Cd, cadmium; Cu, copper; Hg, mercury; Mn, manganese; Pb, lead; Se, selenium; Zn, zinc.

Table S3. Multiple linear regression of maternal elements levels and birth outcomes. Changes in birth outcome per unit increase in ln-transformed elements concentrations (μg/L) in the overall samples are presented.

Elements	Gestational age[a] (n = 594)		Birth weight[b] (n = 595)		Birth length[b] (n = 596)		Head circumference[b] (n = 594)	
	Effect estimate[c] (95% CI)	*P* value	Effect estimate[c] (95% CI)	*P* value	Effect estimate[c] (95% CI)	*P* value	Effect estimate[c] (95% CI)	*P* value
Cu	0.65 (0.06-1.23)	**0.030**	-431.89 (-645.36 to -218.42）	**< 0.001**	-1.55 (-2.55 to -0.55)	**0.002**	-1.27 (-2.01 to -0.54)	**0.001**
Mn	0.28 (-0.02 to 0.57)	0.064	32.09 (-76.12 to 140.30)	0.561	-0.64 (-1.14 to -0.13)	**0.013**	0.16 (-0.21 to 0.53)	0.404
Se	0.33 (-0.02 to 0.68)	0.064	-267.74 (-390.73 to -144.74)	**< 0.001**	-1.97 (-2.53 to -1.41)	**< 0.001**	-0.91 (-1.34 to -0.49)	**< 0.001**
Zn	0.22 (-0.35 to 0.79)	0.453	-231.86 (-441.56 to -22.16)	**0.030**	-0.71 (-1.69 to 0.27)	0.153	-0.41 (-1.13 to 0.31)	0.265
As	-0.05 (-0.29 to 0.19)	0.684	2.53 (-87.28 to 92.35)	0.956	0.003 (-0.42 to 0.42)	0.987	-0.17 (-0.4 to 0.13)	0.266
Cd	0.12 (-0.05 to 0.29)	0.176	-3.63 (-67.77 to 60.52)	0.912	-0.21 (-0.51 to 0.09)	0.161	-0.21 (-0.42 to 0.01)	0.066
Hg	0.15 (0.03-0.28)	**0.013**	-46.26 (-91.17 to -1.35)	**0.044**	-0.39 (-0.60 to 0.19)	**< 0.001**	-0.18 (-0.33 to -0.03)	**0.021**
Pb	0.25 (0.07-0.43)	**0.006**	-65.65 (-131.31 to 0.02)	0.050	-0.61 (-0.92 to -0.31)	**< 0.001**	-0.31 (-0.53 to -0.08)	**0.007**

Bold *p* values are statistically significant ($p < 0.05$)

[a] Gestational age model was adjusted for maternal age, parity, pre-pregnancy BMI, smoking, education and birth weight and birth length.

[b] Models were adjusted for maternal age, parity, pre-pregnancy BMI, smoking, education and gestational age.

[c] Effect estimates using unstandardized β and 95% confidence interval.

Abbreviations: As, arsenic; Cd, cadmium; Cu, copper; Hg, mercury; Mn, manganese; Pb, lead; Se, selenium; Zn, zinc.

Table S4. Regional-specific multiple linear regression of maternal elements levels and birth outcomes. Changes in birth outcome per unit increase in ln-transformed elements concentrations (μg/L) in Ushuaia and Salta samples are presented.

Elements	**Gestational age**[a]				**Birth weight**[b]			
	Ushuaia		Salta		Ushuaia		Salta	
	Effect estimate[c] (95% CI)	*P* value	Effect estimate[c] (95% CI)	*P* value	Effect estimate[c] (95% CI)	*P* value	Effect estimate[c] (95% CI)	*P* value
Cu	0.45 (-0.50 to 1.41)	0.352	0.54 (-0.22 to 1.30)	0.166	-238.45 (-567.26 to 90.36)	0.154	-496.18 (-780.38 to -211.99)	**<0.001**
Mn	0.06 (-0.39 to 0.52)	0.783	0.32 (-0.09 to 0.73)	0.124	87.29 (-68.68 to 243.27)	0.271	55.22 (-101.29 to 211.73)	0.488
Se	1.07 (0.14-2.00)	**0.025**	-0.46 (-1.10 to 0.17)	0.151	-305.55 (-631.04 to 19.94)	0.066	-422.81 (-660.58 to -185.03)	**0.001**
Zn	0.49 (-0.48 to 1.47)	0.318	0.43 (-0.34 to 1.20)	0.269	-92.14 (-431.19 to 246.91)	0.592	-488.49 (-774.64 to -202.34)	**0.001**
As	0.09 (-0.27 to 0.45)	0.614	-0.12 (-0.45 to 0.22)	0.499	36.65 (-88.30 to 161.60)	0.563	-50.92 (-180.02 to 78.18)	0.439
Cd	0.23 (-0.05 to 0.50)	0.106	0.0004 (-0.20 to 0.23)	0.997	24.90 (-71.65 to 121.45)	0.611	-6.52 (-93.22 to 80.17)	0.883
Hg	0.10 (-0.12 to 0.32)	0.380	0.13 (-0.03 to 0.29	0.113	18.70 (-57.52 to 94.92)	0.629	-57.06 (-118.22 to 4.10)	0.067
Pb	0.02 (-0.38 to 0.42)	0.934	0.23 (0.01-0.45	**0.045**	122.14 (-14.86 to 259.15)	0.080	-88.90 (-173.69 to -4.11)	**0.040**
Elements	**Birth length**[b]				**Head circumference**[b]			
	Ushuaia		Salta		Ushuaia		Salta	
	Effect estimate[c] (95% CI)	*P* value	Effect estimate[c]	*P* value	Effect estimate[c] (95% CI)	*P* value	Effect estimate[c] (95% CI)	*P* value
Cu	0.03 (-1.48 to 1.54)	0.966	-2.05 (-3.34 to -0.76)	**0.002**	-0.80 (-2.08 to 0.48)	0.221	-1.31 (-2.23 to -0.39)	**0.006**
Mn	-0.30 (-1.01 to 0.41)	0.407	-0.16 (-0.87 to 0.55)	0.656	0.19 (-0.41 to 0.79)	0.527	0.50 (0.001 to 1.01)	0.050
Se	-1.67 (-3.15 to -0.19)	**0.027**	-1.57 (-2.65 to -0.50)	**0.004**	-1.08 (-2.33 to 0.18)	0.092	-0.65 (-1.42 to 0.12)	0.097
Zn	-0.74 (-2.28 to 0.81)	0.347	-2.58 (-3.86 to -1.29)	**<0.001**	-0.88 (-2.18 to 0.43)	0.186	-0.96 (-1.89 to -0.03)	**0.043**
As	-0.08 (-0.65 to 0.49)	0.778	-0.29 (-0.87 to 0.29)	0.330	-0.12 (-0.60 to 0.36)	0.630	-0.33 (-0.74 to 0.09)	0.123
Cd	-0.03 (-0.47 to 0.41)	0.898	-0.22 (-0.61 to 0.17)	0.276	0.01 (-0.37 to 0.38)	0.973	-0.24 (-0.51 to 0.04)	0.094
Hg	-0.12 (-0.46 to 0.23)	0.507	-0.26 (-0.54 to 0.02)	0.064	-0.29 (-0.58 to 0.00)	**0.047**	0.01 (-0.19 to 0.21)	0.935

Pb	0.24 (-0.39 to 0.87)	0.455	-0.46 (-0.85 to -0.08)	**0.018**	-0.16 (-0.69 to 0.37)	0.559	-0.16 (-0.43 to 0.12)	0.259

Bold *p* values are statistically significant ($p < 0.05$)
[a] Gestational age model was adjusted for maternal age, parity, pre-pregnancy BMI, smoking, education and birth weight and birth length.
[b] Model was adjusted for maternal age, parity, pre-pregnancy BMI, smoking, education and gestational age.
[c] Effect estimates using unstandardized β and 95% confidence interval.
Abbreviations: As, arsenic; Cd, cadmium; Cu, copper; Hg, mercury; Mn, manganese; Pb, lead; Se, selenium; Zn, zinc.

Table S5. Results of logistic regression analyses showing the relationships between tertile of blood elements levels and preterm birth and low birth weight.

Essential elements	Tertiles	Preterm birth[a]		Toxic elements	Tertiles	Preterm Birth[a]	
		OR (95% CI)	*P* value			OR (95% CI)	*P* value
Cu	Tertile 1	Reference		As	Tertile 1	Reference	
	Tertile 2	0.95 (0.28-3.24)	0.934		Tertile 2	0.76 (0.2-2.83)	0.679
	Tertile 3	2.04 (0.45-9.23)	0.353		Tertile 3	0.91 (0.24-3.46)	0.887
Mn	Tertile 1	Reference		Cd	Tertile 1	Reference	
	Tertile 2	2.2 (0.57-8.5)	0.250		Tertile 2	1.83 (0.53-6.38)	0.343
	Tertile 3	3.6 (0.73-17.55)	0.115		Tertile 3	4.45 (0.91-21.79)	0.065
Se	Tertile 1	Reference		Hg	Tertile 1	Reference	
	Tertile 2	5.54 (1.01-30.05)	0.050		Tertile 2	1.29 (0.35-4.69)	0.704
	Tertile 3	3.45 (0.68-17.6)	0.137		Tertile 3	1.81 (0.46-7.09)	0.396
Zn	Tertile 1	Reference		Pb	Tertile 1	Reference	
	Tertile 2	1.72 (0.35-8.45)	0.505		Tertile 2	1.24 (0.35-4.4)	0.744
	Tertile 3	0.7 (0.18-2.66)	0.596		Tertile 3	1.26 (0.32-5.00)	0.743

Essential elements	Tertiles	Low birth weight[b]		Toxic elements	Tertiles	Low birth weight[b]	
		OR (95% CI)	*P* value			OR (95% CI)	*P* value
Cu	Tertile 1	Reference		As	Tertile 1	Reference	
	Tertile 2	2.14 (0.3-15.14)	0.444		Tertile 2	0.56 (0.07-4.21)	0.570
	Tertile 3	0.38 (0.06-2.33)	0.296		Tertile 3	0.39 (0.05-3.19)	0.376
Mn	Tertile 1	Reference		Cd	Tertile 1	Reference	
	Tertile 2	2.55 (0.3-21.34)	0.389		Tertile 2	0.28 (0.04-2.06)	0.211
	Tertile 3	0.48 (0.08-2.9)	0.422		Tertile 3	0.17 (0.02-1.54)	0.116
Se	Tertile 1	Reference		Hg	Tertile 1	Reference	
	Tertile 2	0.09 (0.02-1.13)	0.051		Tertile 2	0.51 (0.09-3.03)	0.458
	Tertile 3	0.14 (0.01-1.39)	0.093		Tertile 3	0.55 (0.09-3.22)	0.506
Zn	Tertile 1	Reference		Pb	Tertile 1	Reference	
	Tertile 2	0.27 (0.03-2.15)	0.215		Tertile 2	0.59 (0.10-3.55)	0.566
	Tertile 3	0.44 (0.06-3.05)	0.402		Tertile 3	0.53 (0.09-3.16)	0.488

[a] Models were adjusted for maternal age, parity, smoking, pre-pregnancy BMI, education and low birth weight.

[b] Models were adjusted for maternal age, parity, smoking, pre-pregnancy BMI, education

and preterm birth.

Abbreviations: As, arsenic; Cd, cadmium; Cu, copper; Hg, mercury; Mn, manganese; Pb, lead; Se, selenium; Zn, zinc; OR, Odds ratio; CI, Confidence interval.

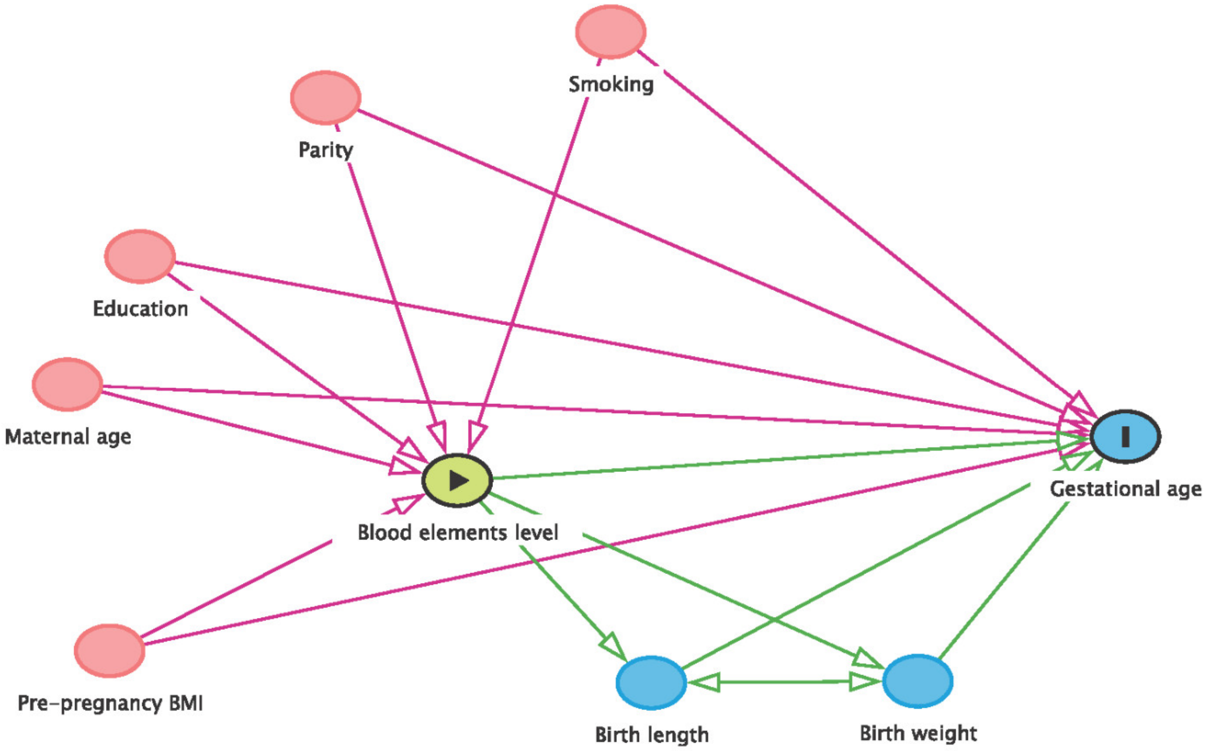

Figure S1. Directed acyclic graph for gestational age model.

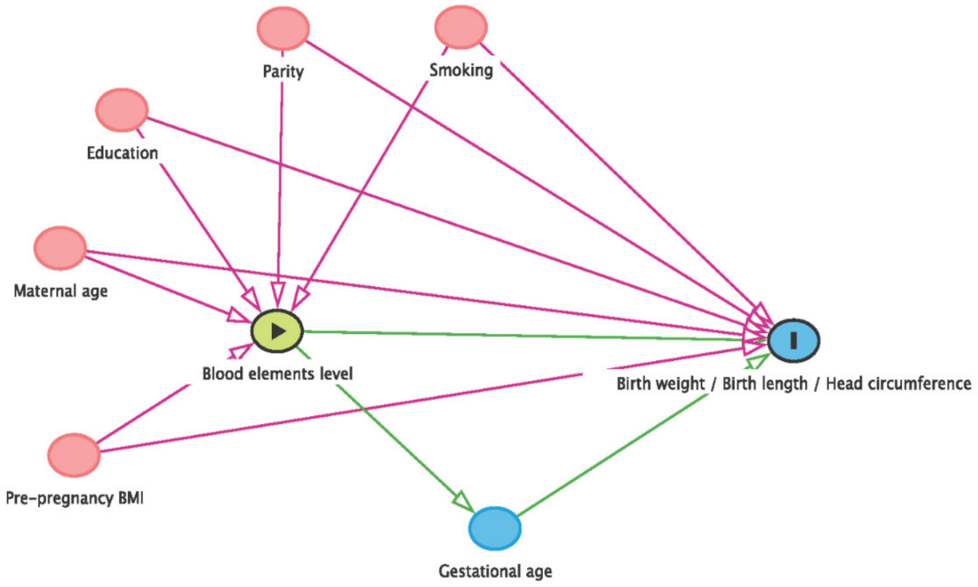

Figure S2. Directed acyclic graph for birth weight, birth length and head circumference models.

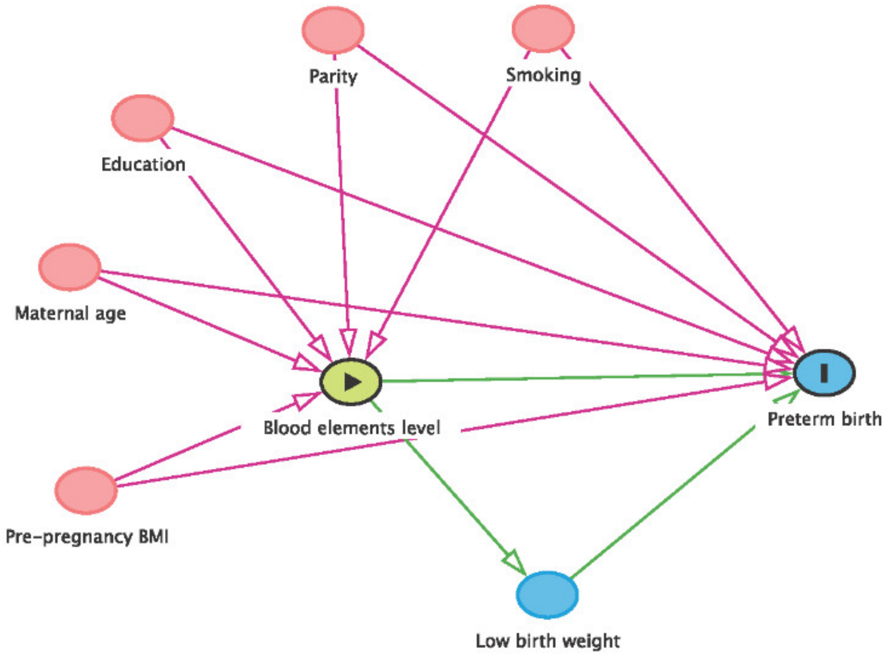

Figure S3. Directed acyclic graph for preterm birth model.

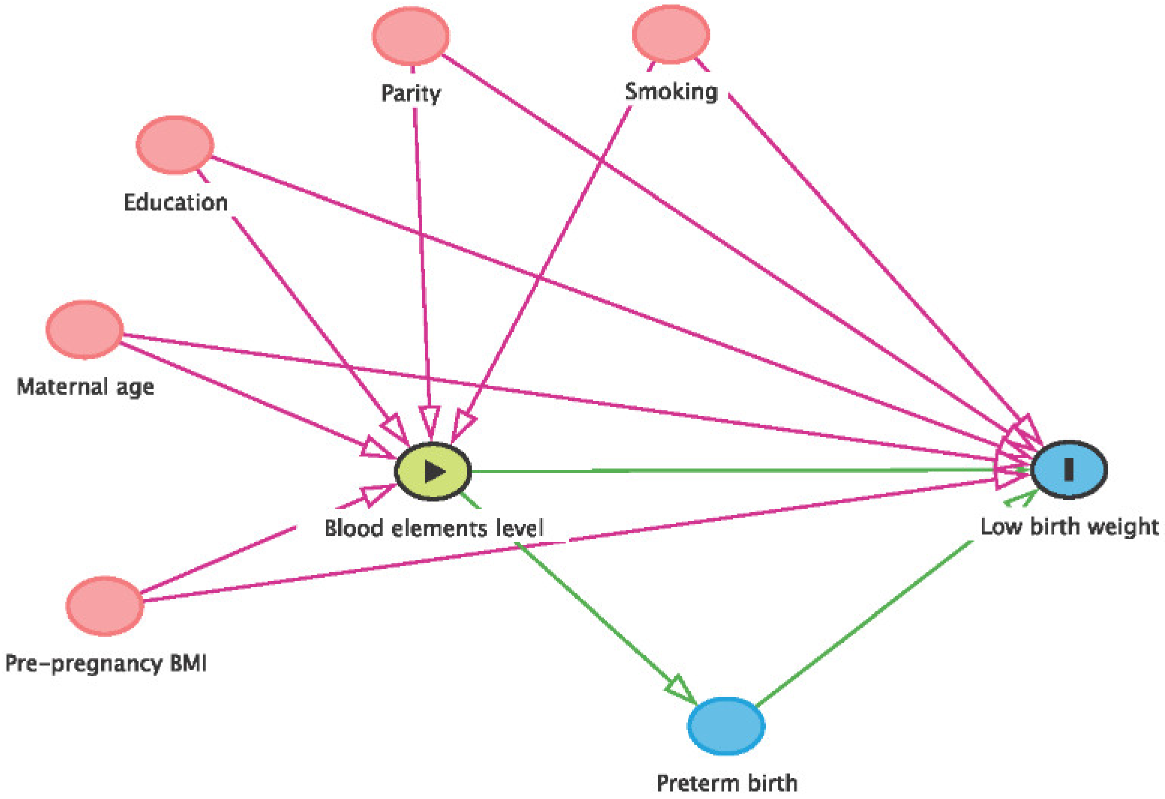

Figure S4. Directed acyclic graph for low birth weight model.

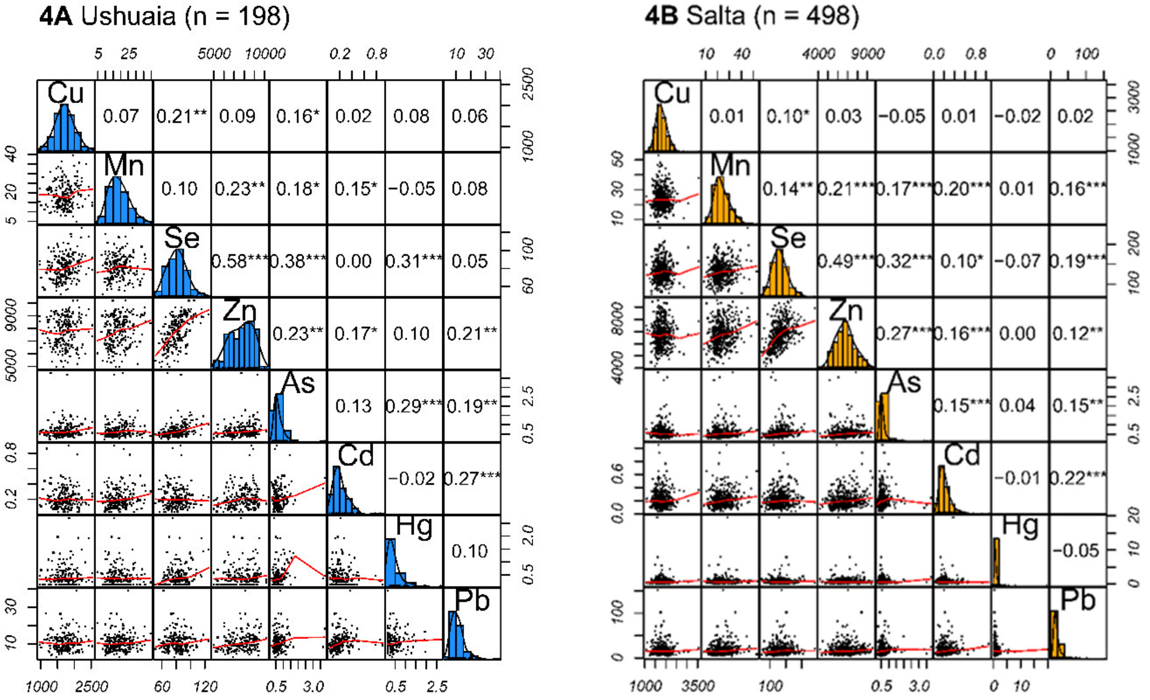

Figure S5. Spearman's correlation coefficients (ρ) among the whole blood toxic and essential elements stratified by study sites. *** $p < 0.001$, ** $p < 0.01$, * $p < 0.05$.

Abbreviations: As, arsenic; Cd, cadmium; Cu, copper; Hg, mercury; Mn, manganese; Pb, lead; Se, selenium; Zn, zinc.

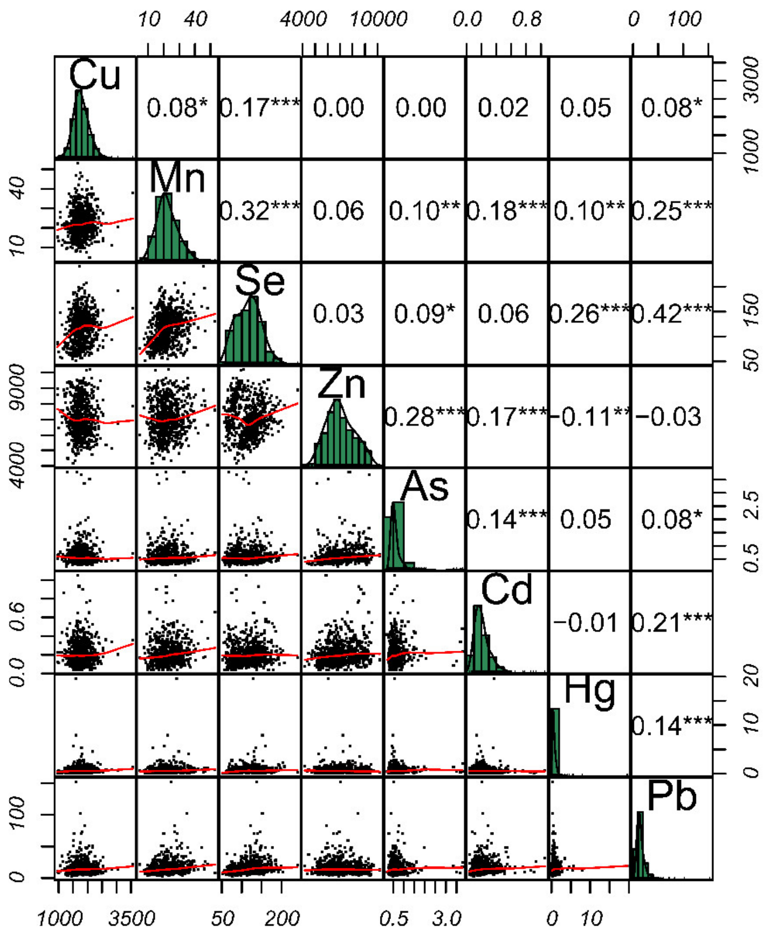

Figure S6. Spearman's correlation coefficients (ϱ) between maternal elements concentrations among the overall postpartum women in the EMASAR study (n = 696). $^{***}p < 0.001$, $^{**}p < 0.01$, $^{*}p < 0.05$.

Abbreviations: As, arsenic; Cd, cadmium; Cu, copper; Hg, mercury; Mn, manganese; Pb, lead; Se, selenium; Zn, zinc.
